# A systematic review of compliance with indoor tanning legislation

**DOI:** 10.1186/s12889-018-5994-4

**Published:** 2018-10-04

**Authors:** Jessica Reimann, Jennifer E. McWhirter, Andrew Papadopoulos, Cate Dewey

**Affiliations:** Department of Population Medicine, Ontario Veterinary College, University of Guelph, Guelph, Ontario N1G 2W1 Canada

**Keywords:** Indoor tanning, Legislation, Compliance, Enforcement, Health policy

## Abstract

**Background:**

Many jurisdictions have enacted indoor tanning legislation in response to the health risks of artificial ultraviolet (UV) radiation exposure. Key components of these legislations include banning minors’ access, requiring parental consent or accompaniment, providing protective eyewear, posting health warning signs, and communicating important health risk information. However, legislation must be complied with to be impactful. Evidence around compliance with indoor tanning legislations has not been synthesized and is an important step toward determining changes in practice due to legislation.

**Methods:**

A systematic review was conducted to obtain peer-reviewed literature about compliance with indoor tanning legislation worldwide. Six databases were searched, resulting in 12,398 citations. Sixteen studies met the inclusion criteria (peer-reviewed scientific studies, published in English, focused primarily on compliance with indoor tanning legislations, and focused on commercial indoor tanning in indoor tanning facilities).

**Results:**

Compliance with most aspects of indoor tanning legislation varied widely. There was good compliance for provision of protective eyewear (84 to 100%; mean = 92%; SD = 8). Compliance with age restrictions ranged from 0 to 100% (mean = 65%; SD = 25), while compliance with posting warning labels in the required locations within a tanning facility ranged from 8 to 72% (mean = 44%; SD = 27). Variation in compliance may be due to true differences, study methodology, or temporal trends.

**Conclusions:**

Variability in compliance with indoor tanning legislation, as found in this systematic review, indicates the legislations may not be having their intended protective effects on the public’s health. The reasons for such low and varied compliance with certain aspects of legislation, and high compliance with other aspects of legislation, deserve further attention in future research to inform best practices around ensuring high and consistent compliance with indoor tanning legislations worldwide.

**Electronic supplementary material:**

The online version of this article (10.1186/s12889-018-5994-4) contains supplementary material, which is available to authorized users.

## Background

The incidence of skin cancer is increasing [1]. One in every three cancers diagnosed worldwide is a form of skin cancer [2]. Approximately 2 to 3 million cases of non-melanoma skin cancers (NMSC) and 132,000 cases of melanoma skin cancer occur globally each year [3]. Ultraviolet (UV) radiation is the main risk factor for skin cancer [4]. Artificial UV radiation exposure from indoor tanning (IT) is responsible for an increasing number of skin cancers [5] and, unlike solar UV exposure, is an entirely avoidable type of UV exposure.

IT is common in North American and most European countries, especially among female young adults and adolescents [5]. This trend is a concerning public health issue as approximately 450,000 cases of non-melanoma skin cancers per year and 10,000 cases of melanoma skin cancers per year in Europe, Australia, and the US combined are attributable to IT [6]. Exposure to IT is associated with a 29% and 67% increased risk of basal cell carcinoma and squamous cell carcinoma, respectively [7]. Importantly, the risk of lifetime melanoma skin cancer increases by 59% with use of IT devices before the age of 35 [8]. This risk is greatest for those 20–29 years of age [9]. Excessive artificial UV radiation can also lead to premature ageing of the skin (wrinkling, age spots, loss of collagen), eye disease (cataracts, ocular melanoma), and immune suppression [10, 11]. Given these dangers, the World Health Organization’s International Agency for Research on Cancer (IARC) classifies UV radiation from IT beds as a Group 1 carcinogen, in the same category as smoking tobacco and asbestos [12].

Numerous countries have implemented IT legislation, focusing especially on banning minors’ access to protect the health of the public. France was the first country to ban youth under the age of 18 from IT in 1997, with Brazil enacting similar legislation in 2002 [13]. Since then, several countries have followed, and some have passed even more stringent access legislations. For example, in 2011 Brazil banned IT for all age groups, and in 2015 Australia banned commercial tanning salons [13, 14]. At the time of writing, Canada, the United States (US), Australia, European countries, including France and Germany, and South American countries, including Chile, have enforceable IT legislation. These legislations include banning minor access, requiring parental consent or accompaniment, requiring protective eyewear, posting of warning signs, and communicating important health risk information. In the US specifically, 44 states and the District of Columbia have enforceable IT legislation, including restricting access to and use of IT facilities by minors [15]. Additionally, the Food and Drug Administration (FDA) and Federal Trade Commission (FTC) regulate IT at the Federal level, through labelling and manufacturing of IT devices, and prohibiting false or misleading health claims about IT device use [16].

Legislation has the power to influence social norms, beliefs, and health risk behaviours [17–19]. It is one of the most powerful policy tools available to governments, and is the most widely used [20]. An effective enforcement program is required to ensure any regulation meets its intended impact [21]. While studies have been published on compliance with IT legislation, the results have not been synthesized. To address this research gap, we conducted a systematic review to evaluate the compliance with IT legislations around the world.

## Methods

### Search strategy

Following PRISMA guidelines (Additional file 1) [22], a systematic review of business, medical, policy, and psychology databases was conducted in November 2016 to obtain peer-reviewed literature about compliance with IT legislations worldwide. Databases were chosen based on their coverage of relevant subject matter. Search terms were generated using the topic of the review, keywords from known relevant studies, MeSH terms, and database thesauri. Search terms were grouped by themes and combined using appropriate Boolean operators. The search terms for IT included*: indoor tanning, artificial tanning, suntan, tanning bed, sunbed, sunbathing, sunlamp, tanning facilities, solarium, tanning device.* The policy-related search terms were: policy, policies, legislat*, regulat*, act, bill, law, ban, restrict, enforce, control, compliance, government legislations, license, licensure, national health policy, youth access, adolescent access, minor, evaluation. The skin cancer-related search terms were: *melanoma, skin cancer, skin neoplasm, basal cell carcinoma, squamous cell carcinoma, malignant melanoma, and cutaneous melanoma*. IT search terms or skin cancer search terms were combined with policy search terms to retrieve all articles relating to IT and policy or skin cancer and policy. The databases searched, and the number of results returned from each, were: PubMed (*n* = 6447), Medline (*n* = 5241), JSTOR (*n* = 133), ABI/INFORM (*n* = 149), Business Source Complete (*n* = 197), PsycINFO (*n* = 230). In total, 12,398 studies were found: 5492 were duplicates, resulting in 6906 unique studies to be screened. Reference lists from relevant studies were also searched for additional studies to include; however, this process did not identify any new studies that the database search had not already identified.

### Selection criteria

**Fig. 1:**
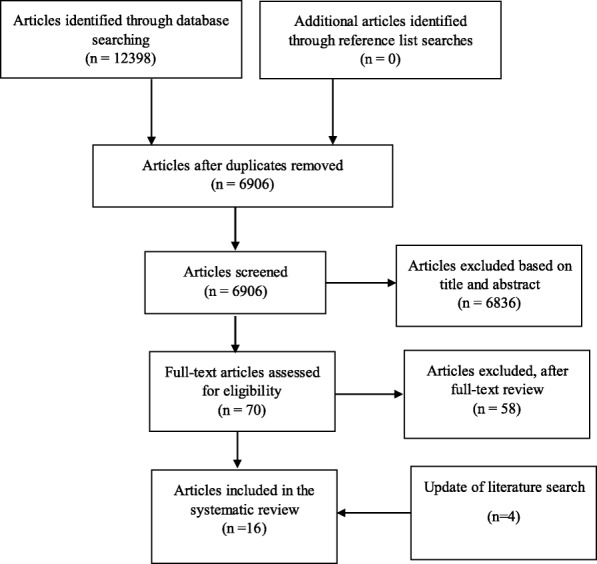
Flow Chart for search and selection of studies of compliance with indoor tanning policy

To be included in this systematic review, studies had to be peer-reviewed scientific studies, published in English, focused primarily on IT legislation (compliance with of legislations, not voluntary guidelines), and focused on commercial IT in IT facilities. There were no restrictions regarding year or country. Compliance was defined broadly by the authors as the criteria provided by the included studies, in relation to fulfilling the requirements of the legislation of interest for each included study (whether Federal/National, or State level). The exclusion criteria were systematic reviews or commentary style studies, grey literature, studies about spray/lotion/solar tanning, and studies about the impact of IT legislation on youth IT. Impact of IT legislation was defined as studies describing the change in prevalence and frequency of IT attributable to the implementation of IT legislation restricting youth access. After applying inclusion and exclusion criteria, 6836 studies were excluded based on title and abstract screening. Another 58 studies were excluded after full text screening. Overall, 12 studies met all inclusion criteria and were thus included in the review. The authors returned to the literature in June 2018 to check for additional studies to be included. Web of science was used to search for studies citing those already included in the review. This uncovered four additional studies, for a total of 16 studies included in this review. Figure 1 outlines the process of exclusion of studies based on exclusion criteria.

Two additional files present the critical appraisal as supplementary information. Additional file 2 provides supplementary information for the critical appraisal tool used. Each study, critical appraisal question, and the resulting score can be found in Additional file 3.

### Data extraction

The first author conducted data extraction. The information extracted from each study included the following: author names, title, date of publication, country, population or site of interest, study design, sample size, outcome(s) of interest, and key findings relevant to compliance and enforcement of IT legislation. These results were grouped by aspect of the legislation they dealt with. A summary of this information appears in Table 1. Additionally, means and standard deviations were calculated where possible.

**Table 1:** Compliance with Indoor Tanning Legislation Outcomes of Interest

First Author (Year)	Country (State)	Date of Data Collection	Legislation^a^	Methods	Site of Study	Outcomes of Interest
Brouse (2011) [37]	US (NY)	2010	Federal	In-person observation of the facility (*N* = 224 IT beds in 85 facilities)	Individual IT beds within IT facilities	Warning Labels: 65% of IT beds had warning labels present, 14% had warning labels that were barely visible, 24% were moderately visible, 25% were clearly visible, and 1% were completely visible.
Choy (2017) [23]	US (14 states)	2015	State	Underage telephone shoppers (*N* = 412)	IT facilities and their operators	Age: 80% of facilities complied with under 17 or under 18 age restrictions. Health Effects: 20% of operators reported skin cancer, 52% reported sunburn, and 4% reported premature ageing. 10% denied any dangers from IT. Health Benefits: 89% of operators reported specific health benefits.
Culley (2001) [33]	US (CA)	1998	State and Federal	In-person underage shoppers with observation of the facility (*N* = 54)	IT facilities and their operators	Parental Consent: 43% of facilities required parental consent for ages 14–18. Eye Protection: 100% of facilities provided protective eyewear, 89% required protective eyewear. Warning Labels: 85% of facilities had warning labels present, 74% had warning labels accessible, legible, correct, 85% had other (exposure) labels present, 74% had other (exposure) labels accessible, legible, correct, 20% had a warning sign posted in the IT area, and 15% had a warning sign that was legible, accessible, and correct. Health Effects: 32% of operators reported skin cancer, and 98% reported skin burns.
De Maleissye (2011) [38]	France	2009	National	Online website observation (*N* = 71)	IT facility websites	Warning Labels: 35% of websites mentioned the ‘black box’ legal warning. Health Beneifts: 7% of websites reported health benefits.
Fleischer (1993) [34]	US (NC)	1991	State and Federal	Facility inspection by researchers (*N* = 32)	IT facilities	Parental Consent: 13% of facilities had a minor consent form available and in use. Eye Protection: 84% of facilities had protective eyewear available. Warning Labels: 78% of facilities had warning signs that were easily viewed by customers, 72% had a warning sign posted within 1 metre of IT stations, 90% had warning sign text that was compliant, and 78% had warning labels attached to the sunlamp. Health Effects: 19% of facilities had a consumer statement about risks available for customers to sign.
Forster (2006) [30]	US (MN, MA)	Not Reported	State	In-person underage shoppers (*N* = 200 facilities × 2 = 400 facility visits), followed up by telephone interviews (*N* = 136)	IT facilities and their operators	Age: By telephone, 19% of operators reported having a minimum age requirement; In person, 31% of operators did not sell an IT session to an underage buyer, 60% of operators assessed age eligibility, 57% asked for age, but did not assess identification, and 3% checked identification. When age was not asked and identification was not checked, a purchase attempt was successful 98% of the time, when age was asked but identification was not checked, a purchase attempt was successful 50% of the time, and when identification was checked, a purchase attempt was successful 35% of the time. Parental Consent: By telephone, 87% of operators complied with parental consent. In person, 32% of operators complied with parental consent.
Gorig (2018) [36]	Germany	2015	National	Telephone interviews (*N* = 357)	Individuals who had used IT facilities since 2012	Eye Protection: 87% of sunbed users were provided with protective eyewear, 85% were advised to use protective eyewear, and 68% used protective eyewear during their last sunbed use. Health Effects: 57% of sunbed users were given the opportunity to determine their skin type. 43% of sunbed users were ever advised of the negative health effects of IT, while 33% were ever offered written risk information.
Grewal (2013) [24]	US (CA)	2013	State	Underage telephone shoppers (*N* = 338)	IT facilities and their operators	Age: 77% of facility operators complied with under 18 age restrictions. Health Effects: 16% of operators reported skin cancer, 11% reported sunburn, and 2% reported premature ageing. 61% of operators denied any dangers from IT. Health Benefits: 72% of operators reported specific health benefits. Risk Restrictions: 59% of operators stated daily IT was acceptable, and 22% of operators stated that unlimited IT was acceptable.
Heilig (2005) [40]	US (CO, IL, TX, WI)	2003	State	Underage telephone shoppers (*N* = 400)	IT facilities and their operators	Health Effects: 54% of operators reported skin cancer (42 to 81%), 87% of operators reported sunburn (76 to 93%), and 54% of operators reported premature ageing (41 to 79%).
Hester (2005) [25]	US (CO, IL, TX, WI)	2003	State	Underage telephone shoppers (N = 400)	IT facilities and their operators	Age: 23% of operators in TX, 74% of operators in IL, and 89% of operators in WI complied with under 13 age restrictions. 77% of operators in WI complied with under 16 age restrictions. Parental Consent: 74% of operators complied with parental consent in IL, 6% complied with parental accompaniment in TX.
Hurd (2006) [35]	US (CA)	2004	State	Underage telephone shoppers and in-person underage shoppers (*N* = 115)	IT facilities and their operators	Parental Consent: By telephone, 73% of operators complied with parental consent. In person, 64% of operators complied with parental consent.
Makin (2011) [31]	Australia (Victoria)	2009	State	Underage telephone shoppers and in-person underage shoppers (*N* = 30)	IT facilities and their operators	Age: By telephone, 23% of operators inquired about the customer’s age and 10% informed them identification was required. In person, when age was concealed, 80% of underage research assistants were granted access by operators, and 3% were allowed access who openly disclosed their age. Eye Protection: 97% of facilities provided eyewear. Warning Labels: 97% of facilities displayed the mandatory warning sign with the risk of skin cancer. Health Effects: 10% of operators reported skin cancer as a risk over the telephone, and 97% reported skin cancer in-person. Risk Restrictions: 90% of facilities complied with minimum time between exposures, 53% complied with skin type restrictions, 87% complied with whether they conducted a skin type assessment, and 83% complied with customer consent forms.
Pichon (2009) [26]	US (50 states)	2006	State	Underage telephone shoppers (*N* = 3647 facilities)	IT facilities and their operators	Age: 70% of operators complied with under 16 age restrictions in Wisconsin. Parental Consent: 93% of operators complied with parental consent, and 43% complied with parental accompaniment.
Salomone (2009) [29]	Chile	2008	National	In-person underage shoppers with observation of the facility (*N* = 24 facilities)	IT facilities and their operators	Age: 62% of facilities complied with stating under 18 age limits. Parental Consent: 50% of facilities complied with parental consent. Eye Protection: 25% of facilities complied with compulsory use of goggles. Warning Labels: 8% complied with use of obligatory warning sign in the reception, 63% had a warning sign in the IT booth, while 29% of the centers had no warning signs. Health Effects: 46% of operators gave oral spontaneous information, 0% of facilities had written information, 25% of operators reported potential risks, and 19% of facilities displayed a list of photosensitizing agents. Health Benefits: 29% of operators reported health benefits.
Tripp (2017) [27]	US (TX)	2015	State	Underage telephone shoppers (*N* = 635)	IT facilities and their operators	Age: 81% of facilities complied with under 18 age restriction.
Williams (2018) [28]	US (42 states and the District of Columbia)	2015–2016	State	Underage telephone shoppers (*N* = 427)	IT facilities and their operators	Age: Percent of operators complying with age restrictions by state: Under 14: GA (50%), ID (10%), ME (70%), ND (70%), WV (20%) Under 15: AL (0%) Under 16: PA (70%), WI (80%) Under 17: CT (50%), NJ (70%), NY (70%) Under 18: CA (90%), DE (90%), DC (50%), HI (86%), IL (100%), LA (70%), MN (90%), NV (60%), NH (100%), NC (50%), OR (100%), TX (80%), VT (70%), WA (60%) Parental Consent: Percent of operators complying with parental consent by state: Under 15: VA (70%) Under 18: AZ (90%), AR (40%), MA (70%), MI (30%), MS (40%), OH (90%), RI (70%), SC (90%) Percent of operators complying with parental accompaniment by state: Under 14: KY (30%), MA (70%), MS (40%), TN (40%) Under 15: WY (30%) Under 16: NB (70%), IN (50%) Under 18: UT (90%)

*AL* Alabama, *AR* Arkansas, *AZ* Arizona, *CA* California, *CO* Colorado, *CT* Connecticut, *DC* District of Columbia, *DE* Delaware, *FL* Florida, *GA* Georgia, *HI* Hawaii, *ID* Idaho, *IL* Illinois, *LA* Louisiana, *MA* Massachusetts, *MD* Maryland, *ME* Maine, *MI* Michigan, *MN* Minnesota, *MS* Mississippi, *NC* North Carolina, *ND* North Dakota, *NH* New Hampshire, *NJ* New Jersey, *NV* Nevada, *NY* New York, *OH* Ohio, *OR* Oregon, *PA* Pennsylvania, *RI* Rhode Island, *SC* South Carolina, *TN* Tennessee, *TX* Texas, *UT* Utah, *VA* Virginia, *VT* Vermont, *WA* Washington, *WI* Wisconsin, *WV* West Virginia

^a^Language is consistent with what was found in the study with respect to the legislation. Detailed legislative requirements can be found in Table 2

## Results

### Study characteristics

An overview of the general study characteristics and key study outcomes can be found in Table 1. Most studies were conducted in the US (*n* = 12), with the others conducted in Germany (*n* = 1), France (*n* = 1), Australia (*n* = 1), and Chile (*n* = 1). Of those conducted in the US, they most commonly examined legislation in one state (*n* = 6), two states (*n* = 1), and four states (*n* = 2), while one study evaluated legislation compliance in 14 states, one evaluated 42 states and the District of Columbia, and one evaluated all 50 states. Specific legislative requirements examined in the included studies can be found in Table 2.

**Table 2:** Relevant Legislations Examined in the Included Studies

First Author (Year)	Country (State)	Relevant Legislation (Year of Implementation)	Legislation Requirements Examined in the Study
Brouse (2011) [37]	US (NY)	21 Code of Federal Regulations (CFR) 1040.20 (1985)	Each sunlamp product must have a warning label^b^ The warning label must be “permanently affixed or inscribed on an exterior surface of the product when fully assembled for use so as to be legible and readily accessible to view by the person being exposed immediately before the use of the product.”
Choy (2017) [23]	US (14 states)	FTC: Indoor Tanning State of California, Section 22706 of the Business and Professions Code (2012) Connecticut General Statutes Section 19a-232 (2012) DC Act 20–549 (2014) Delaware Code Chapter 30D (2015) Hawaii Revised Statutes 321–12.2 (2015) Illinois Part 795 Tanning Facilities Code (2014) Louisiana State Legislature Act 193 (2014) Minnesota statutes. Regulation of tanning facilities. 325H.0858 (2014) Nevada Revised Statute 597.7617 (2013) New York Public Health Law 3555 (2012) Oregon Health Authority Public Health Division Chapter 333–119-0090 (2013) Tex. Health and Safety Code Ann. 145.008 (2013) Vermont 18 V.S.A. 1513 (2012) Washington State Legislature Chapter 18.370 Tanning Facilities (2013)	Ban under 17: CT, NY Ban under 18: CA, DE, DC, HI, IL, LA, MN, NV, OR, TX, VT, WA Prohibit false or misleading health claims about IT
Culley (2001) [33]	US (CA)	Filante Tanning Facility Act (1988) 21 Code of Federal Regulations (CFR) 1040.20 (1985)	Parental consent for ages 14–18 Protective eyewear provided and required for facility use Warning sign posted in tanning area Warning sign legible, accessible, correct^b^ Danger labels present Danger statement legible, accessible, correct Other (exposure) labels present Other (exposure) labels legible, accessible, correct
De Maleissye (2011) [38]	France	Decret no 97–617 relatif a` la vente et a` la mise a` disposition du public de certains appareils de bronzage utilisant des rayonnements ultraviolets. (1997)	‘Black box’ legal warning: ‘Artificial ultraviolet radiation may damage the skin and eyes. These biological effects depend on the type and intensity of the radiation dose and on individual skin sensitivity (skin phototype)’. Claiming any beneficial health effect of IT is forbidden
Fleischer (1993) [34]	US (NC)	15A NCAC, Section 1400 (1990) 21 Code of Federal Regulations (CFR) 1040.20 (1985)	Minor consent form for parental/guardian signature available and in use Protective eyewear available and compliant Ultraviolet light warning signs easily viewed by customer, posted within 1 m of tanning stations, and text compliant with statute Equipment compliant with federal regulations, has product labels^b^ Consumer statement outlining risks available for customer to sign
Forster (2006) [30]	US (MN, MA)	Massachusetts statutes. Tanning facilities. 105 SMR Vol 123 (1994) Minnesota statutes. Regulation of tanning facilities. 325H (1993)	Parental consent through signing a required warning statement in person, witnessed by an employee, before the initial tanning session (16 years in MN, 14–17 years in MA) Parental accompaniment required under 14 in MA
Gorig (2018) [36]	Germany	Regulation of hazardous artificial ultraviolet radiation (2012)	Provide and require use of protective eyewear Determine the skin type of the customer Provision of information on the hazards and health risks of exposure to ultraviolet radiation (in oral and written formats)
Grewal (2013) [24]	US (CA)	State of California, Section 22706 of the Business and Professions Code (2012)	Ban under 18 Must sign a statement with acknowledgment of risks Ban of claims that state IT is safe or have any known health benefits Limited exposure times
Heilig (2005) [40]	US (CO, IL, TX, WI)	Colorado Department of Public Health and the Environment. Artificial tanning device regulations (Section 25–5-106) (1989) Illinois Department of Public Health. Tanning facilities code (77 Ill. Adm. Code 795) (1992) Texas Department of Health. Rules for licensure of tanning facilities (25 Texas Administrative Code, 229.341–357) (2002) Wisconsin Statutes & Annotations. Chapter 255: Chronic disease and injuries (s. 255.08) (2001)	Required to give copy of warning statement (not signed): CO Require a signed warning statement: IL, TX, WI
Hester (2005) [25]	US (CO, IL, TX, WI)	Illinois Department of Public Health. Tanning facilities code (77 Ill. Adm. Code 795) (1992) Texas Department of Health. Rules for licensure of tanning facilities (25 Texas Administrative Code, 229.341–357) (2002) Wisconsin Statutes & Annotations. Chapter 255: Chronic disease and injuries (s. 255.08) (2001)	Ban under 13: TX Ban under 14: IL Ban under 16: WI Parental consent 14–17 in IL, and 16–17 in TX Parental accompaniment 13–15 in TX
Hurd (2006) [35]	US (CA)	Filante Tanning Facility Act (1988)	Parental consent under 18 Parental accompaniment under 14
Makin (2011) [31]	Australia (Victoria)	Victorian Government. Radiation Amendment (Tanning Units and Fees) Under section 139 of the Radiation Act (2008)	Ban under 18 Protective eyewear must be worn Require a signed warning statement which says that exposure to UV radiation contributes to skin cancer Set a minimum of 48 h between exposures Ban individuals with skin type 1
Pichon (2009) [26]	US (50 states)	State level legislation for the included states (States with youth access legislation as of 2006)^c^	Ban under 16: WI Parental consent: AZ, CA, FL, GA, IL, IN, LA, ME, MA, MI, MN, MS, NH, NC, OH, OR, RI, SC, TN, TX Parental accompaniment: IN, TX
Salomone (2009) [29]	Chile	Reglamento de Solariums o Camas Solares. Decreto No. 70/06 (2007)	Age limits must be stated Parental consent under 18 Provide and require the use of protective eyewear Warning signs must be present in the reception and tanning areas Require a signed warning statement about the risks of IT
Tripp (2017) [27]	US (TX)	Tex. Health and Safety Code Ann. 145.008 (2013)	Ban under 18
Williams (2018) [28]	US (42 states and DC)	State level legislation for the included states (States with youth access legislation as of 2015/2016)^c^	Ban under 14: GA, ID, ME, ND, WV Ban under 15: AL Ban under 16: PA, WI Ban under 17: CT, NJ, NY Ban under 18: CA, DE, DC, HI, IL, LA, MN, NV, NH, NC, OR, TX, VT, WA Parental consent under 15: VA Parental consent under 18: AZ, AR, MD, MI, MS, OH, RI, SC Parental accompaniment under 14: KY, MA, MS, TN Parental accompaniment under 15: WY Parental accompaniment under 16: NB, IN Parental accompaniment under 18: UT

*AL* Alabama, *AR* Arkansas, *AZ* Arizona, *CA* California, *CO* Colorado, *CT* Connecticut, *DC* District of Columbia, *DE* Delaware, *FL* Florida, *GA* Georgia, *HI* Hawaii, *ID* Idaho, *IL* Illinois, *LA* Louisiana, *MA* Massachusetts, *MD* Maryland, *ME* Maine, *MI* Michigan, *MN* Minnesota, *MS* Mississippi, *NC* North Carolina, *ND* North Dakota, *NH* New Hampshire, *NJ* New Jersey, *NV* Nevada, *NY* New York, *OH* Ohio, *OR* Oregon, *PA* Pennsylvania, *RI* Rhode Island, *SC* South Carolina, *TN* Tennessee, *TX* Texas, *UT* Utah, *VA* Virginia, *VT* Vermont, *WA* Washington, *WI* Wisconsin, *WV* West Virginia

^b^This regulation requires each sunlamp product to have a label that contains a warning statement with the words: “DANGER — Ultraviolet radiation. Follow instructions. Avoid overexposure. As with natural sunlight, overexposure can cause eye and skin injury and allergic reactions. Repeated exposure may cause premature aging of the skin and skin cancer. WEAR PROTECTIVE EYEWEAR; FAILURE TO MAY RESULT IN SEVERE BURNS OR LONG-TERM INJURY TO THE EYES. Medications or cosmetics may increase your sensitivity to the ultraviolet radiation. Consult physician before using sunlamp if you are using medications or have a history of skin problems or believe yourself especially sensitive to sunlight. If you do not tan in the sun, you are unlikely to tan from the use of this product”

^c^Relevant legislations for studies with more than 15 states are not listed. To access a detailed list of US legislations, please visit http://www.ncsl.org/research/health/indoor-tanning-restrictions.aspx

All the studies used observational, cross-sectional designs (*n* = 16). The studies focused on IT facilities, their operators, IT users, and other aspects of the IT business. The most common location or population of interest was IT facility operators (*n* = 12), with the remaining studies focused on IT facilities (*n* = 1), IT users (*n* = 1), individual IT beds (*n* = 1), and IT facility websites (*n* = 1). Sample sizes varied widely by study: IT facility operators (*n* = 24 to *n* = 3647); IT users (*n* = 357) IT facilities (*n* = 32); IT beds (*n* = 224 devices from *n* = 85 facilities); and IT facility websites (*n* = 71).

A variety of methods for investigating compliance were used in the studies, alone and in various combinations, but most commonly included telephone or in-person “secret shopper” strategies. These strategies included research assistants posing as potential clients in the following combinations: underage telephone secret shoppers (*n* = 7), underage telephone secret shoppers plus underage in-person secret shoppers (*n* = 2), underage in-person secret shoppers with facility observation (*n* = 2), underage in-person secret shoppers with follow-up telephone interview (*n* = 1), in-person facility observation by researchers (*n* = 1), online website observation (*n* = 1), and facility inspection (*n* = 1). Additionally, one study interviewed IT users on the telephone.

### Study outcomes

The outcomes of the 16 studies are grouped into the following compliance categories: age restriction (*n* = 9), parental consent or accompaniment (*n* = 8), protective eyewear (*n* = 5), warning labels (*n* = 6), health risk information (*n* = 8), health benefit information (*n* = 4), and risk restrictions (*n* = 2). We summarize the findings for each of these outcomes below. Table 3 highlights the means and ranges of percent compliance for each outcome.

**Table 3:** Ranges and Means of Compliance for Key Outcomes of Interest

Outcome	Range (%)	Mean (%)	Standard Deviation	Number of Studies	Studies (First Author, Date)	Locations
Age						
Under 13	23–89	62	35	1	Hester, 2005	IL, TX, WI
Under 14	10–70	44	28	1	Williams, 2018	GA, ID, ME, ND, WV
Under 16	70–80	74	5	3	Hester, 2005; Pichon, 2006; Williams, 2018	PA, WI
Under 17 or 18	20–100	72	22	6	Choy, 2017; Forster, 2006; Grewal, 2013; Makin, 2011; Tripp, 2017; Williams, 2018	CA, CT, DC, DE, HI, IL, LA, MA, MN, NC, NH, NJ, NV, NY, OR, TX, VT, WA, Australia
Overall	0–100	65	25	9	Choy, 2017; Forster, 2006; Grewal, 2013; Hester, 2005; Makin, 2011; Pichon, 2006; Salomone, 2009; Tripp, 2017; Williams, 2018	AL, CA, CO, CT, DC, DE, GA, HI, ID, IL, LA, MA, ME, MN, NC, ND, NH, NJ, NV, NY, OR, PA, TX, VT, WA, WI, WV, Australia, Chile
Telephone	0–100	65	25	8	Choy, 2017; Forster, 2006; Grewal, 2013; Hester, 2005; Makin, 2011; Pichon, 2006; Tripp, 2017; Williams, 2018	AL, CA, CO, CT, DC, DE, GA, HI, ID, IL, LA, MA, ME, MN, NC, ND, NH, NJ, NV, NY, OR, PA, TX, VT, WA, WI, WV, Australia
In Person	20–62	34	24	3	Forster, 2006; Makin, 2011; Salomone, 2009	MA, MN, Australia, Chile
Parental Consent						
Overall	13–93	62	24	8	Culley, 2001; Fleischer, 1993; Forster, 2006; Hester, 2005; Hurd, 2006; Pichon, 2009; Salomone, 2009; Williams, 2018	AR, AZ, CA, CO, FL, GA, IL, LA, MA, MD, ME, MI, MN, MS, NC, NH, OH, OR, RI, SC, TN, TX, VA, WI, Chile
Telephone	30–93	71	21	5	Forster, 2006; Hester, 2005; Hurd, 2006; Pichon, 2009; Williams, 2018	AR, AZ, CA, CO, FL, GA, IL, LA, MA, MD, ME, MI, MN, MS, NH, OH, OR, RI, SC, TN, TX, VA, WI
In Person	13–64	40	19	5	Culley, 2001; Fleischer, 1993; Forster, 2006; Hurd, 2006; Salomone, 2009	CA, NC, MA, MN, Chile
Parental Accompaniment						
Overall (Telephone)	6–90	47	24	3	Hester, 2005; Pichon, 2009; Williams, 2018	ID, IN, KY, MA, MS, NB, TN, TX, UT, WY
Eyewear^d^						
Availability and Provision	84–100	92	8	3	Culley, 2001; Fleischer, 1993; Makin, 2011	CA, NC, Australia
Required Use	25–89	57	45	2	Culley, 2001; Salomone, 2009	CA, Chile
Warning Labels						
Location Compliance	8–97	60	29	5	Brouse, 2011; Culley, 2001; De Maleissye, 2011; Fleischer, 1993; Makin, 2011; Salomone, 2009	CA, NC, NY, Australia, Chile, France
Content Compliance	15–90	63	33	2	Culley, 2001; Fleischer, 1993	CA, NC
Health Effects						
Overall	0–98	45	31	7	Choy, 2017; Culley, 2001; Fleischer, 1993; Grewal, 2013; Heilig, 2005; Salomone, 2009	CA,CO, CT, DC, DE, HI, IL, LA, MN, NC, NV, NY, OR, TX, VT, WA, WI, Chile
General Question	2–52	18	18	2	Choy, 2017; Grewal, 2013	CA, CT, DC, DE, HI, IL, LA, MN, NV, NY, OR, TX, VT, WA
Explicit Question	32–98	65	24	2	Culley, 2001; Heilig, 2005	CA, CO, IL, TX, WI
Skin Cancer	10–97	43	29	5	Choy, 2017; Culley, 2001; Grewal, 2013; Heilig, 2005	CA, CO, CT, DC, DE, HI, IL, LA, MN, NV, NY, OR, TX, VT, WA, WI
Sunburn	11–98	73	31	4	Choy, 2017; Culley, 2001; Grewal, 2013; Heilig, 2005	CA, CO, CT, DC, DE, HI, IL, LA, MN, NV, NY, OR, TX, VT, WA, WI
Premature Ageing	2–79	37	29	3	Choy, 2017; Grewal, 2013; Heilig, 2005	CA, CO, CT, DC, DE, HI, IL, LA, MN, NV, NY, OR, TX, VT, WA, WI
Health Benefits						
Health Benefits Claimed^e^	7 – 89^e^	49^e^	38	4	Choy, 2017; De Maleissye, 2011; Grewal, 2013; Salomone, 2009	CA, CT, DC, DE, HI, IL, LA, MN, NV, NY, OR, TX, VT, WA, France, Chile

^d^Gorig, 2018 was not included in the calculation of means, since individuals who use tanning facilities were surveyed, rather than the people running the facilities or the facilities themselves. Doing so allowed for the denominator (tanning facilities/operators) to be consistent

^e^Health benefits claimed are reported as non-compliance. Studies reported the number of facilities who claimed health benefits even though legislation does not allow health benefit claims. All other outcomes are reported as compliance

#### Age restriction

Nine studies investigated compliance with age restrictions by noting if IT facilities state age restrictions verbally and adhere to them. Age compliance was evaluated using underage telephone secret shopper requests to buy IT services [23–28], underage in-person secret shopper requests [29], or both [30, 31].

In two studies, minimum age requirements in IT facilities were evaluated. In Chile, 62% of IT facility operators reported having a minimum age requirement (telephone inquiry) [29]. In Minnesota and Massachusetts, 19% of IT facilities self-reported serving minors, regardless of stated age restrictions (in-person inquiry) [30]. For both studies, perfect compliance would be 100% of the facilities having and following the minimum age requirement.

Eight studies investigated compliance with specific age restrictions (i.e., 13, 14, 15, 16, 17, 18 years of age), six via telephone, and two via in-person inquiries. In three US states (Illinois, Texas, and Wisconsin) with an under 13 age restriction, compliance ranged from 23 to 89% (telephone inquiry) [25]. In this case, operators reported that they would not permit someone under 13 to tan. In five US states with an under 14 age restriction (Georgia, Indiana, Maine, North Dakota, and West Virginia), compliance ranged from 10 to 70% (telephone inquiry) [28]. In one US state with an under 15 age restriction (Alabama), no IT facilities complied [28]. Compliance with an under 16 age restriction was 70% [26], 77% [25] and 80% [28] in one US state (Wisconsin) (telephone inquiry). Additionally, in another US state with an under 16 age restriction (Pennsylvania), compliance was 70% [28]. Legislation restricting access to either those under 17 or 18 years of age was complied with by 80% of IT facilities across 14 US states, when the operator was asked if the underage caller could use the IT facilities (telephone inquiry) [23]. More specifically, legislation with an under 17 age restriction in three US states (Connecticut, New Jersey, and New York) ranged from 50 to 70%, and legislation with an under 18 age restriction in 14 states (California, Delaware, DC, Hawaii, Illinois, Louisiana, Minnesota, Nevada, New Hampshire, North Carolina, Oregon, Texas, Vermont, Washington) ranged from 50 to 100% [28]. Additionally, legislation prohibiting those under 18 years of age was complied with by 77% [24] of IT operators in California (telephone inquiry), 81% of IT facilities in Texas (telephone inquiry) [27], 31% [30] of IT facilities in Minnesota and Massachusetts (in-person inquiry), and 20% [31] of IT facilities in Australia (in-person inquiry).

Two studies investigated compliance with age inquiries and requests for age identification. An Australian study using telephone inquiry found 23% of IT operators inquired about the customer’s age and 10% informed them age identification was required [31]. In person, 77% of Australian IT operators inquired about the customer’s age, and 17% asked for age identification [31]. Overall, 80% of operators allowed an underage shopper to tan if age was concealed, and 3% of underage shoppers were allowed to tan who openly disclosed their age [31]. A study in Minnesota and Massachusetts [30] using in-person inquiry found that 60% of operators assessed age eligibility, 57% inquired about age, but did not assess age identification, and 3% assessed age identification. Age inquiries and requests for age identification proved important for whether a minor was able to make a successful purchase in this study: when operators did not inquire about age and identification was not assessed, a purchase attempt was successful 98% of the time; when operators inquired about age but identification was not assessed, a purchase attempt was successful 50% of the time; and when identification was assessed, a purchase attempt was successful 35% of the time [30].

Temporal lapse, the time between when legislation was enacted and when compliance was evaluated, was considered in the context of age restriction compliance. When the time lapse between passing legislation and measuring compliance was one to two years, compliance was lower (*n* = 3; 20% to 77%; mea*n* = 46%, SD = 28) than when the time lapse was 11 to 14 years (n = 4; 70% to 89%; mean = 77%, SD = 7). However, the mean for compliance of age restrictions at one to two years post-legislation may be biased by one study that investigated compliance as a recent update to a law that had already been in place for 25 years [32]. Upon update of the literature and the inclusion of a new study which is the largest to date (44 states), and most recent study published at the time of writing, this temporal relationship did not remain [28].

#### Parental consent or accompaniment

Eight studies investigated compliance with parental consent or accompaniment requirements [25, 26, 28–30, 33–35]. Seven of these were conducted in the US and evaluated state-level legislation; one was conducted in Chile and evaluated national legislation.

Compliance with parental consent aspects of legislations ranged from 13 to 93%. This varied by assessment method: higher compliance was reported via telephone, (30 to 93%) [25, 26, 28, 30, 35]; lower compliance was reported with in-person visits (13 to 64%) [29, 30, 33–35]. When the time lapse between passing legislation and checking compliance was one to two years compliance was lower (6% to 50%; *n* = 3; mean = 23%, SD = 23) than when the time between was 11–14 years (32 to 87%; *n* = 3; mean = 64%, SD = 14).

Compliance with parental accompaniment was investigated in three US studies via telephone. In Texas 6% of operators complied with parental accompaniment legislation [25]. In a study of Indiana and Texas, conducted four years later, 43% of facilities complied [26]. In a study conducted more recently, 30 to 70% of operators complied with under 14 parental accompaniment legislation (Kentucky, Massachusetts, Mississippi, and Tennessee), 30% of operators complied with an under 15 parental accompaniment legislation (Wyoming), 50 and 70% of operators complied with under 16 parental accompaniment legislations (Indiana and Nebraska), and 90% of operators complied with an under 18 parental accompaniment legislation (Utah) [28].

#### Eye protection

Four studies investigated compliance with the availability and/or provision of protective eyewear through in-person inquiries at IT facilities. One additional study asked IT users about their experiences with eye protection [36]. Two of these studies were conducted in the US [33, 34], one in Australia [31], one in Chile [29], and one in Germany [36]. Most (84 to 100%) IT facilities provided protective eyewear as required by the legislation [31, 33, 34]. When IT users themselves were asked, 87% reported they had been provided with protective eyewear, while 85% reported they were advised to use protective eyewear [36]. This legislation requires the provision and recommended use of protective eyewear [36]. Additionally, individual states have their own protective eyewear compliance rules (see Table 2 for details). Three studies evaluated whether facilities were compliant with requiring clients to use the provided protective eyewear. Of the IT facilities providing protective eyewear in California, 89% required the use of that protective eyewear [33]. In contrast, even though legislation in Chile stipulates both provision and mandatory use of protective eyewear, 25% of IT facilities in Chile made the use of protective eyewear mandatory [29]. Additionally, when IT users were asked, 68% had actually used protective eyewear during their last IT [36].

#### Warning labels

Compliance with displaying required warning labels varied widely among the six studies using in-person inquiries [29, 31, 33, 34, 37, 38]. Compliance with sign location varied from 8 to 97%. In Chile, 8% of IT facilities had an obligatory sign in the reception area, 20% had a warning sign posted in the IT area, and 63% had a sign in the IT booth [29]. Legislation in Chile stipulates that signs must be visible in the IT facility reception and in IT service areas [29]. In the US, the FDA requires a clearly visible warning sign on each IT bed [39]. Three US studies observed warning labels on 65% [37], 78% [34], and 85% [33] of IT beds; but, even when warning labels were observed, there were problems with their visibility. Twenty-five percent of IT beds had warning labels that were “clearly visible” and 1% of IT beds had warning labels that were “completely visible” [37]. Additionally, 78% of warning signs were easily viewed by customers and 72% of warning signs were posted within 1 m of IT stations [34]. In Australia, 97% of IT facilities displayed mandatory warning signs indicating skin cancer risk [31]. One study investigated warning statements on IT facility websites; 35% of French websites complied with the legislative requirement to include France’s black box legal warning [38].Two studies in the US assessed compliance of the text content of warning labels. FDA warning label content requirements can be found in Table 2. A study in North Carolina found 90% of warning signs had text that was compliant with federal legislation [34]. A study in California found that of IT facilities, 15% had warning signs that were correct (as well as accessible and legible), 74% had danger labels that were correct (as well as accessible and legible), and 74% had exposure labels that were correct (as well as accessible and legible) [33].

#### Health risk information

Seven studies reported compliance with the provision of health risk information by IT facility operators, using in-person methods [29, 33, 34], telephone methods [23, 24, 40], or both [31]. One additional study reported compliance with health risk information through telephone interviews with IT users [36].

In addition to the required posting of warning labels containing health risk information, health risk information is also legally required in oral or written formats depending on the jurisdiction. Three studies evaluated compliance with written health risk information: in one, 19% of IT facilities had a consumer statement about risks available for customers to sign, as required by state legislation [34]; and in another, 0% provided written information about IT beds, which the IT facilities are required to provide to customers [29]. In the third study, 33% of IT users were ever offered written health risk information [36]. With respect to compliance with oral information, 61% of operators denied any dangers from IT booths when asked [24], which is in conflict with the legislation from California stating IT facilities “shall not claim, or distribute promotional materials that claim, that using an ultraviolet tanning device is safe or free from risk or that indoor tanning has any known health benefits” [32]. A more recent study, across multiple US states, found 90% of operators did not deny the dangers of IT [23]. Additionally, 43% of IT users were ever advised of negative health risks of IT by operators [36].

Compliance with the provision of specific types of risk information (i.e., skin cancer, sunburn, premature ageing) was assessed in five studies. In US states, when asked explicitly about skin cancer, an average of 49% of IT facility operators reported that skin cancer was a potential health risk of IT [33, 40]. In US states, when asked general, non-specific questions about health risks, an average of 18% of IT facility operators reported that skin cancer was a potential health risk [23, 24]. One Australian study evaluated whether operators reported skin cancer as a risk both on the telephone and in-person: 10% of operators mentioned skin cancer as a risk over the telephone, while 97% mentioned skin cancer in-person [31]. In US states, when asked explicitly about sunburns, an average of 89% of IT facility operators reported that a sunburn was a potential health risk of IT [33, 40]. In US states, when asked about general, non-specific health risks, an average of 32% of IT facility operators reported that a sunburn was a potential health risk [23, 24]. In US states, when asked explicitly about premature ageing, an average of 54% of IT facility operators reported that premature ageing was a potential health risk of IT [40]. In US states, when asked about general, non-specific health risks, an average of 3% of IT facility operators reported that premature ageing was a potential health risk [23, 24].

#### Health benefit information

Four studies reported non-compliance with legislation prohibiting beneficial health claims. In general, false, and misleading health claims about the health benefits of IT are prohibited by IT legislations. In the US, the FTC mandates that IT facilities must avoid all claims that suggest a health benefit of IT [16]. During in-person inquiries, 72% of IT facility operators in the US [24] and 29% [29] of IT facility operators in Chile promoted IT as healthy. On the telephone, 89% of IT facilities in the US claimed false and misleading health benefits, which are prohibited by the legislation under study [23]. In France, claiming any beneficial health effects of IT is forbidden, and 7% of IT facility websites did not comply with legislation, by mentioning supposed beneficial health effects of IT [38].

#### Risk restrictions

Two studies evaluated compliance with state-specific legislations regarding exposure schedules and skin type [24, 31]. In direct conflict with US FDA exposure schedules, in California 59% of IT facility operators stated that daily IT was acceptable and 22% of IT facility operators stated that unlimited IT was acceptable [24]. In Australia, legislation mandates a minimum of 48 h is required between IT exposures; however, customers with fair skin that burns easily (“Type 1”) are banned from IT [41]. Although 90% of operators complied with minimum time requirements between IT sessions when asked, 47% of fair-skinned secret shoppers were granted access to an IT facility [31].

## Discussion

In this systematic review of 16 studies across four countries, compliance with IT legislation varied. Although the studies indicated relatively high (92% on average) and somewhat consistent compliance for the provision of protective eyewear, there was variability and suboptimal compliance for other components of legislation. For example, compliance with warning signs was lower (60% on average), and compliance with age-restrictions was much lower (34% on average with in-person methods). Variability may be due to true differences, or methodological, jurisdictional, or temporal factors. IT legislation is clearly not meeting its intended outcome of total compliance. We can, however, use lessons learned from other successful health legislations to suggest areas for improvement. The most effective strategy may be through youth-focused and knowledge-based approaches, along with the use of effective enforcement.

### Youth access

The elevated skin cancer risk to young people has been a major impetus for the implementation of legislation to restrict the age of those using IT devices [42]. The long-term risks of melanoma associated with artificial UV radiation exposure at young ages is a serious public health problem [3, 8]. However, compliance with age restrictions and parental consent varied, and during in-person inquiries was, on average, very low.

Greater efforts around enforcement of youth access legislation are necessary to reduce the prevalence of IT among youth. Stronger public health interventions are needed to address the significant health and economic burden of youth IT [19]. As with other risky behaviours, IT often begins during adolescence [43], and youth targeted interventions have been successful with regard to reducing other voluntary risk behaviours, including tobacco use. Restricting youth access to tobacco has been an important component of tobacco legislation [44]. Prohibiting tobacco sales to youth, conducting unannounced inspections, and raising the legal purchasing age, have significantly decreased youth tobacco sales [19]. Indeed, raising the legal tobacco purchasing age above 18 or 19 to 21 is seen as a favourable way to prevent youth tobacco use [45]. IT legislation should mandate an age restriction of at least 18, and possibly higher than 18, and the mandatory checking of age identification as it increases compliance with age restrictions [30, 31]. Relatedly, it is likely that checking age identification of IT facility customers who appear to be under 25 would reduce minor access to IT facilities. This would be similar to what is used to restrict the sale of tobacco and alcohol to minors, where age identification requests have been shown to reduce sales [44]. Unlike tobacco or alcohol, IT cannot be purchased by someone else and provided to a minor. Therefore, enforcement of age bans, and age identification checks should be more successful in reducing minor access to IT facilities.

Differences in compliance across studies with respect to age and parental consent may be due to different methodological approaches across studies. On average, just over two-thirds of IT facility operators complied with age restrictions when contacted by telephone, but only one-third did so in-person. Similarly, two-thirds of IT facility operators complied with parental consent over the telephone, but less than half did so with in-person inquiries. There was higher reported compliance with telephone methods and lower compliance with in-person “secret shopper” methods. Hence, compliance with IT legislation estimated by telephone methods may be overestimated. In-person methodologies may more closely resemble real-life scenarios and may provide a more accurate reflection of true compliance with IT legislation, while social acceptability bias impacts telephone methodologies. Although two of the studies commented that telephone methods and in-person methods are similar in their accuracy of evaluation of compliance [26, 35], the results of this review suggest otherwise. One exception was the study by Hurd et al. (2006); however, for both the in-person and telephone methods in that study, the IT facility operator was prompted with a question. All studies using telephone methodologies used prompting when asking about age or parental consent. Such direct questions about age compliance or parental consent do not necessarily directly measure, or accurately reflect, the business behaviour (i.e., selling IT sessions to minors).

Compliance appeared to vary with the time between when legislation was passed and when compliance was examined. Compliance with youth access aspects of IT legislation increased as time from enactment to evaluation increased. When the time lapse between passing a law, and measuring compliance was two years or less, compliance with age and parental consent was lower than when the time lapse was more than 10 years. Such temporal trends were also reported with smaller time differences (e.g., less than one year, one to two years, two or more years) [23]. These differences may have occurred because operators may take more time to become aware of, and comply with, new legislations, or enforcement may not occur promptly following the enactment of new legislations.

Upon our update to the literature and the inclusion of newly published studies, the temporal relationship between the implementation of IT legislation and when research was conducted became less clear. Rather, there may be a relationship between the overall number of jurisdictions with legislation and higher compliance, even with short time lapses between implementation and evaluation. Perhaps as more jurisdictions implement IT legislation, compliance with new legislation occurs more quickly, as these types of restrictions are expected by the IT facilities and their clients. There may have previously been a temporal relationship with those jurisdictions first adopting IT legislations, but with time this has lessened. Even the results from the 44-state study conducted in 2018 did not show a temporal relationship between implementation and evaluation of IT legislation [28], while the 14-state study conducted in 2017 explicitly discussed the presence of a temporal relationship [23]. This difference may have occurred because more states are adopting IT legislation in the US and around the world, or because the number of states included in the two studies differed, among other possible reasons.

There were insufficient studies from countries other than the US to evaluate between-country differences; however, we have noted some jurisdictional trends in findings from the US studies. There are regional differences in compliance with IT legislation across the US. When compliance across multiple US states was investigated, relative to other states with similar legislation, states in the south reported lower compliance with IT legislation for youth access [23, 25, 28]. It is unclear why this is the case, but could be due to differences in climate, political and social environment, or state differences in legislation or enforcement. Regarding the latter, for example, legislation regarding youth access in Texas outlines strict enforcement, but penalties are less severe than in other states [25].

### Risk communication

Effective health communication is an important tool used by public health to alter risk behaviours. It is important for individuals to be aware of exposure to health risks, especially if the risk is harmful, yet avoidable. This research examined compliance with communication-related aspects of IT legislation, including communicating health risks, use of warning labels, and risk restrictions. Knowledge of the risks of IT can allow customers to make informed decisions; however, the public lacks knowledge and understanding of IT risks [46, 47]. Providing health risk information, not using misleading health benefit claims, enforcing risk restrictions, and posting informative warning signs, are all important ways to ensure the communication of appropriate and correct health and risk information.

The provision of health risk information varied, as did using misleading health benefit claims. The combination of insufficient risk information communicated appropriately, and IT facility operators claiming health benefits from IT will lead to wholly misinformed customers. In two studies evaluating risk information, IT facility operators were either asked about general health risks [24], or specifically about skin cancer and sunburn [33]. Compliance rates were higher when operators were asked about specific health problems relative to general health problems, but even then, less than half of IT facility operators warned of skin cancer as a health risk, while nearly three quarters warned of sunburn. Possible explanations may include that operators are not knowledgeable about the health risks of IT [24, 40], or they may fear they are deterring potential customers and selectively choose to communicate risk information. However, we do not know how customers are asking about risk information, and therefore are unable to determine if they are receiving specific and appropriate health risk information.

Warning labels are an important method of communicating health risk information. In the context of other health risk behaviours besides IT, they raise awareness of avoidable health issues, influence health behaviours, and even support other aspects of related health policies [48–50]. In addition, health warning labels increase conversations about risky behaviour, and can shift social norms about these health behaviours [51]. Given the low compliance with the provision of health risk information at IT facilities, the communicative role of warning labels is heightened. Compliance with location and content of warning labels varied and, on average, was poor. Two-thirds of IT facility operators complied with both warning sign location and the content needed on those warning signs. While both were suboptimal, average compliance was higher for warning label content than location.

Warning label compliance ranges were narrower for federal US legislation (65–85%) [33, 34, 37] than US state-level warning label legislation (20–90%) [33, 34], suggesting a trend by scope of jurisdiction. The US FDA requires IT bed manufacturers to permanently attach federal warning statements to beds during assembly [16, 39, 52]. In contrast, state-level IT facility warning labels differ between states and must be affixed, and sometimes even created by operators, as is the case in California [16, 32]. In comparison, manufacturers are required to include warning labels on cigarette packaging before they can be provided for sale [53] leading to high compliance with warning label requirements for tobacco.

### Protective eyewear

Compliance was high for the provision of protective eyewear, with all studies reporting over 80% compliance, rendering it rather anomalous relative to all other IT legislation components investigated. Eye protection is important because artificial UV radiation can cause acute eye damage and ocular melanoma [54, 55]. Explanations for this high compliance include that it is relatively easy to implement, is low cost, and it likely has little or no negative impact on business because individuals can still tan. It is also possible protective eyewear may be an additional revenue stream for IT facility operators. Some states in the US require IT facilities to provide free eyewear, while others allow for the sale of eyewear [16].

Although a high percentage of IT facilities provided protective eyewear, there was lower compliance with requiring clients to wear the provided protective eyewear [29, 33, 36]. Although it may be relatively easy to provide protective eyewear to clients, it is difficult to ensure the use of eyewear because it involves checking on the client as they enter the IT bed. More research is needed to investigate the extent to which clients are wearing what is provided with respect to eyewear, and whether provision and use are closely correlated.

### Policy implications and recommendations

The variation in compliance, and relatively low compliance, with most aspects of IT legislation, leads to concerns about enforcement. One possible reason why low compliance was reported could be due to low enforcement. Some studies have shown variability in inspection and enforcement practices by health inspectors [56, 57]. Reduction of harm from IT beds for all individuals, including youth, cannot be fully realized without proper enforcement [58]. To increase compliance, an increased level of inspection and enforcement is imperative. This has been seen with enforcement of tobacco legislation [44]. A universal IT tax is one way to fund IT facility inspections [59]. Furthermore, if IT clients are required to pay a higher tax percent, this could become a deterrent to IT use. IT legislation without enforcement, including penalties, is not expected to lead to change.

Overall, greater provisions for enforcement of IT legislation are needed, as without enforcement, compliance is unlikely to improve. Optimal compliance with all areas of IT legislation will likely require increased inspection, and mandatory and stricter penalties for infractions. Increased inspection could be funded by moneys collected through a federal IT tax or an IT business license, which are both already used in some jurisdictions [59]. Protection of youth from the dangers of IT could be improved by mandatory age identification checks, and age identification checks should encompass ages higher than the minimum identified by the legislation. Further, parental consent compliance was low and thus does little to protect youth. We therefore suggest there be no parental consent exceptions, and that all clients under the minimum age be refused service, as is the case in the context of tobacco control. Compliance with warning labels was also suboptimal. In addition to enhanced inspection and penalties, standardization of warning label content and provision of warning labels to IT facility operators may also increase compliance. Further, health “benefit” information should be more widely and aggressively restricted through IT legislation, and the provision of health risk information through other means in addition to warning labels ought to be considered. A multi-pronged approach to risk communication, as used in alcohol and tobacco control, may be more effective. Finally, given the discrepancies in findings between methodological approaches, we suggest policy makers consider in-person checks be considered a best practice in the evaluation of IT legislation.

### Limitations

Only English-language, peer-reviewed studies were included, meaning studies in other languages, and those in the grey literature, were excluded. One author conducted all data extraction. We restricted the review to assess compliance and not impact. Compliance levels inform public health practitioners and policy makers about IT legislations and are a necessary first step. We restricted the review to compliance with legislation and not with voluntary guidelines, because the latter shows poor compliance [31, 60, 61]. Due to differences in legislation and how each study operationalized compliance, the definition of compliance with legislations of interest varied between studies. Further, the broader heterogeneity of studies including variable study designs, temporal and geographical differences, and the different study sites (IT facilities, websites, IT beds, public health inspectors) made comparisons across studies challenging.

### Future research

Given the lower compliance with in-person inquiries compared to telephone inquiries, we encourage researchers to use in-person data collection techniques, which may more accurately reflect day-to-day business practices. The variability in compliance suggests that high-compliance for key aspects of IT legislation is possible, as was noted in some studies. More carefully determining the variables that contribute to high compliance with IT legislation is a priority area for future research, as such findings could inform best practices. Future research should strive to explicitly operationalize compliance, to allow for clearer understanding of research findings.

Future research should also consider the temporal relationship between the implementation of IT legislation, and the timing of compliance research. Researchers may wish to explore how compliance with legislation in a jurisdiction changes over time to further describe temporal trends, which should clarify some of difference in compliance, and shed some light on how long it takes for a legislation to become impactful. Most studies on compliance were conducted in the US, suggesting a need for studies from more countries with different IT legislation experiences. Research is also needed to evaluate why regional differences exist.

Additionally, in-depth case study evaluations identifying factors contributing to successful compliance with and enforcement of a specific jurisdiction’s IT legislation (e.g., process and implementation evaluations) may lead to a more robust understanding of the hindrances and facilitators to high compliance. An understanding of these differences might shed light on ways to improve compliance through legislative amendments.

## Conclusions

The results of this review demonstrate variable and suboptimal compliance with IT legislation. Compliance variability could be partly explained by methodology (in-person vs. telephone), temporal considerations (time proximity to enactment of legislation), level of legislation (federal vs. state), and aspect of legislation (e.g., protective eyewear vs. age restrictions). Compliance with provision of protective eyewear was relatively high, though still imperfect. Importantly, compliance was low for all other key aspects of IT legislation. This was especially true when focused on results from in-person compliance checks, including the highly important age restrictions, as well as for parental consent, warning labels, and health risk and benefit information. Greater compliance is required for youth access, and more effective risk communication is needed, which can be accomplished through increased enforcement and legislative amendments. Future IT policy research and practice endeavours should consider successful strategies from other public health initiatives, such as alcohol and tobacco control.

## Additional files

Additional file 1: PRISMA Checklist. Checklist items indicating the content included in the systematic review, and where each item can be found. (DOCX 20 kb)

Additional file 2: Information for Critical Appraisal. Supplementary information for the critical appraisal tool used. (DOCX 17 kb)

Additional file 3: Critical Appraisal. Study, critical appraisal question, and the resulting score. (DOCX 23 kb)

## Abbreviations

AL: Alabama
AR: Arkansas
AZ: Arizona
CA: California
CO: Colorado
CT: Connecticut
DC: District of Columbia
DE: Delaware
FDA: Food and Drug Administration.
FL: Florida
FTC: Federal Trade Commission.
GA: Georgia
HI: Hawaii
IARC: International Agency for Research on Cancer.
ID: Idaho
IL: Illinois
IN: Indiana
IT: Indoor Tanning
KY: Kentucky
LA: Louisiana
MA: Massachusetts
MD: Maryland
ME: Maine
MI: Michigan
MN: Minnesota
MS: Mississippi
NA: Not Applicable
NB: Nebraska
NC: North Carolina
ND: North Dakota
NH: New Hampshire
NJ: New Jersey
NMSC: Non-Melanoma Skin Cancer
NR: Not Reported
NV: Nevada
NY: New York
OH: Ohio
OR: Oregon
PA: Pennsylvania
RI: Rhode Island
SC: South Carolina
TN: Tennessee
TX: Texas
US: United States
UT: Utah
UV: Ultraviolet
VA: Virginia
VT: Vermont
WA: Washington
WI: Wisconsin
WV: West Virginia
WY: Wyoming
