# Supplementary material for: A systematic review of compliance with indoor tanning legislation
Source: BMC Public Health. 2018 Oct 4;18:1096. doi: 10.1186/s12889-018-5994-4 (PMC6171306; doi:10.1186/s12889-018-5994-4)
Supplement: Supplementary file 2 — Information for Critical Appraisal. Supplementary information for the critical appraisal tool used. (DOCX 17 kb) [file 12889_2018_5994_MOESM2_ESM.docx]

# Additional File 2 –Information for Critical Appraisal

| **Question** | **Explanation** | **Score Assignment** |
| --- | --- | --- |
| 1. Hypothesis | Is the hypothesis/aim/objective of the study clearly described? | No=0  Yes=1 |
| 1. Literature Review | Is the literature review current and complete? | No=0  Partially=0.5  Yes=1 |
| 1. Research Design Identified | Has the research design been clearly identified? | No=0  Partially=0.5  Yes=1 |
| 1. Appropriate Research Design | Is the research design appropriate for the study hypothesis? | No=0  Yes=1 |
| 1. Participants | Is it clear how participants were:   - 1. Initially identified or selected   2. Approached for entry into the study   3. Included or excluded   4. Assessed   5. Confirmed in their assessment | No=0  Partially=0.5  Yes=1 |
| 1. Tabulation of Participants | For each stage of the study, has the number of subjects included or excluded been tabulated? | No=0  Yes=1 |
| 1. Sample Size Calculation | Was the sample size calculation described? Was it appropriate for the methods? Was it adequate for what the researchers were aiming to do?  **NA:** assigned if the study was a census; **NR:** assigned if the sample size calculation was not given | No=0  Yes=1 |
| 1. Ethics | Was the study conducted in an ethical matter? | No=0  Yes=1 |
| 1. Interviewer/Coder Training | Were interviewers/coders trained in collecting data, were they aware of the hypothesis? Were steps taken to reduce interviewer bias?  **NR:** assigned if there was no description of how interviewers were trained | No=0  Partially = 0.5  Yes=1 |
| 1. Structured Data Collection Form | Was a structured data form or survey used, and was the data collected in a similar environment? | No=0  Yes=1 |
| 1. Questions Asked | Have the precise questions asked been reported? | No=0  Partially=0.5  Yes=1 |
| 1. Graded Results | Have the authors described how they graded a result? I.e. Did they explain how or why they coded a result the way they did (especially with interview type questions)? | No=0  Partially=0.5  Yes=1 |
| 1. Confounders | Have confounding or effect-modifying variables been discussed so the reader can judge how they’ve been controlled? | No=0  Partially=0.5  Yes=1 |
| 1. Follow-up | When follow-up occurred, have measurements been described, and adequate to ascertain results aren’t biased? Have all participants been accounted for at follow-up?  **NA:** assigned if there was no follow-up used in the study | No=0  Partially=0.5  Yes=1 |
| 1. Measures of Association | Have correct measures of association been reported?  **NA:** assigned if no measures of association were calculated | No=0  Yes=1 |
| 1. Confidence Intervals | Do measures of association have confidence intervals reported? Are mean values accompanied by a measure of variance?  **NA:** assigned if no measures of association were calculated | No=0  Yes=1 |
| 1. Conclusion Based on Analysis | Is the conclusion based on the analysis? Are the conclusions strongly suggestive when the data more properly suggests a chance finding? Have important observations been ignored? | No=0  Partially=0.5  Yes=1 |
| 1. Explanations for Outcomes | Have competing explanations for the outcomes been discussed? | No=0  Partially=0.5  Yes=1 |
| 1. Statistics | Were all necessary statistics conducted? | No=0  Yes=1 |
| 1. Clinical/Practical Significance | Is clinical/practical significance discussed separately from statistical significance? Are the differences found significant enough to change practices and policies? | No=0  Partially=0.5  Yes=1 |
| 1. Power | Has the power of the study to detect differences been discussed? | No=0  Partially=0.5  Yes=1 |
| 1. Results in the Context of Existing Research | Have study results been placed in the context of existing findings? Have differences with previous work been discussed? Have directions been given for future research? | No=0  Partially=0.5  Yes=1 |
| 1. Policy Implications | If policy implications have been drawn from the data, have they been justified? | No=0  Partially=0.5  Yes=1 |
| 1. Main Findings | Are the main findings of the study clearly described? | No=0  Partially=0.5  Yes=1 |
| 1. P-values | Have actual probability values been reported (i.e. specific p value rather than <0.05)  **NA:** assigned if the study didn’t have statistics to report p-values for | No=0  Yes=1 |
| 1. Representative Participants | Were participants representative of the entire population from which they were recruited? (Whether it be actual individual participants or facilities or websites included) | No=0  Yes=1 |
| 1. Were current policies discussed? | Did the study include comments on the policies in effect at the time of the study? | No=0  Partially=0.5  Yes=1 |
| 1. Was it clear which policy was being evaluated? | Did the authors make it clear which policies were being evaluated for enforcement or compliance? | No=0  Partially=0.5  Yes=1 |
