# Supplementary material for: A systematic review of compliance with indoor tanning legislation
Source: BMC Public Health. 2018 Oct 4;18:1096. doi: 10.1186/s12889-018-5994-4 (PMC6171306; doi:10.1186/s12889-018-5994-4)
Supplement: Supplementary file 3 — Critical Appraisal. Study, critical appraisal question, and the resulting score. (DOCX 23 kb) [file 12889_2018_5994_MOESM3_ESM.docx]

# Additional File 3 - Critical Appraisal

| **First Author** | **Bracken** | | | | | | | | | | | | | | | | | | | | | | | **Downs and Black** | | | **Other** | | **Score**^[[1]](#footnote-1)^ | **Percentage** |
| --- | --- | --- | --- | --- | --- | --- | --- | --- | --- | --- | --- | --- | --- | --- | --- | --- | --- | --- | --- | --- | --- | --- | --- | --- | --- | --- | --- | --- | --- | --- |
|  | **1** | **2** | **3** | **4** | **5** | **6** | **7** | **8** | **9** | **10** | **11** | **12** | **13** | **14** | **15** | **16** | **17** | **18** | **19** | **20** | **21** | **22** | **23** | **24** | **25** | **26** | **27** | **28** |  |  |
| Brouse (2011) | y | y | y | y | y | y | NA | y | y | y | y | y | y | NA | NA | NA | y | n | y | y | n | y | y | y | NA | y | y | y | 21/23 | 91% |
| Culley (2001) | y | y | y | y | y | y | NR | y | y | y | y | y | n | y | y | y | y | y | y | y | y | y | y | y | y | y | y | y | 26/27 | 96% |
| Choy (2017) | y | y | y | y | y | y | NR | y | y | y | y | y | y | NA | NA | NA | y | y | y | y | n | y | y | y | y | y | y | y | 23/24 | 96% |
| De Maleissye (2011) | y | y | y | y | y | y | NR | y | NR | y | y | y | y | NA | NA | NA | y | y | n | y | n | y | y | y | NA | y | y | y | 20/22 | 91% |
| Fleischer (1993) | n | y | y | y | p | n | NR | y | NR | y | y | n | n | NA | NA | NA | y | n | y | y | n | y | p | n | NA | y | y | y | 14/22 | 64% |
| Forster (2006) | y | y | y | y | y | y | NR | y | y | y | p | y | y | y | y | y | y | y | y | y | n | y | y | y | y | y | y | y | 25.5/27 | 94% |
| Gorig (2018) | y | y | y | y | y | y | NR | y | NR | y | p | n | y | y | NA | NA | y | y | y | y | y | y | y | y | NA | y | y | y | 21.5/23 | 93% |
| Grewal (2013) | y | y | y | y | y | y | NR | y | y | y | y | y | y | NA | NA | NA | y | y | y | y | y | y | y | y | y | y | y | y | 24/24 | 100% |
| Heilig (2004) | y | y | y | y | y | y | NR | y | y | y | y | y | n | NA | NA | NA | y | y | n | y | n | p | y | y | NA | y | y | y | 19.5/23 | 85% |
| Hester (2005) | n | y | y | y | y | y | NR | y | y | y | y | n | y | NA | NA | NA | y | y | n | y | n | y | y | y | NA | y | y | y | 19/23 | 83% |
| Hickle (2005) | y | y | y | y | y | y | NA | y | y | y | p | y | y | NA | NA | NA | y | y | y | y | y | y | y | y | NA | y | y | y | 22.5/23 | 98% |
| Hurd (2006) | y | y | y | y | y | y | NR | y | y | y | y | y | y | y | y | y | y | y | y | y | n | y | y | y | NA | y | y | y | 25/26 | 96% |
| Makin (2011) | y | y | y | y | y | y | NR | y | y | y | p | y | n | y | NA | NA | y | n | y | y | y | y | y | y | NA | y | y | y | 21.5/24 | 90% |
| Mayer (2008) | y | y | y | y | y | y | NA | y | y | y | y | y | n | NA | y | n | y | y | y | y | y | y | y | y | y | y | p | p | 23/26 | 88% |
| Pichon (2009) | y | y | y | y | y | y | NR | y | y | y | y | y | y | NA | y | y | y | y | y | y | y | y | y | y | n | y | p | p | 24/26 | 92% |
| Salomone (2009) | y | y | y | y | y | y | NR | y | y | y | y | y | n | NA | NA | NA | y | y | y | y | n | y | y | y | NA | y | y | y | 21/23 | 91% |
| Tripp (2017) | y | p | y | y | y | y | NR | y | NR | y | y | y | y | NA | NA | NA | y | p | y | y | n | y | y | y | NA | y | y | y | 20/22 | 91% |
| Williams (2018) | y | p | y | y | y | n | n | y | NR | y | y | p | y | NA | NA | y | y | y | y | y | n | y | y | y | y | y | y | y | 21/25 | 84% |

Y – yes; n – no; p – partially; NR - not reported; NA – not applicable

1. 1 Denominator was reduced if questions were NA or NR [↑](#footnote-ref-1)
